# Transcriptomics and Metabolomics Analysis of the Ovaries of High and Low Egg Production Chickens

**DOI:** 10.3390/ani12162010

**Published:** 2022-08-09

**Authors:** Xuan Huang, Haiyang Zhang, Haiyue Cao, Wei Zhou, Xin Xiang, Zhaozheng Yin

**Affiliations:** 1 Animal Science College, Zijingang Campus, Zhejiang University, Hangzhou 310058, China; 2 College of Animal Science and Technology, Jiangsu Agri-Animal Husbandry Vocational College, Taizhou 225300, China

**Keywords:** metabolomics, transcriptomics, chicken, ovary, egg production

## Abstract

**Simple Summary:**

The ovarian tissues of different breeds of hens during egg production were investigated through transcriptomics and metabolomics to provide a more comprehensive understanding of the molecular mechanisms of the ovary during egg production. Four genes involved in egg production were predicted by the transcriptome, including P2RX1, INHBB, VIPR2, and FABP3, and several close metabolites associated with reproduction were identified in the metabolome, including 17α-hydroxyprogesterone, iloprost, spermidine and adenosine. Correlation analysis of specific differential genes and differential metabolites identified important gene-metabolite pairs VIPR2–Spermidine and P2RX1–Spermidine in the reproductive process.

**Abstract:**

Egg production is a pivotal indicator for evaluating the fertility of poultry, and the ovary is an essential organ for egg production and plays an indispensable role in poultry production and reproduction. In order to investigate different aspects of egg production mechanisms in different poultry, in this study we performed a metabolomic analysis of the transcriptomic combination of the ovaries of two chicken breeds, the high-production Ninghai indigenous chickens and the low-production Wuliangshan black-boned chickens, to analyze the biosynthesis and potential key genes and metabolic pathways in the ovaries during egg production. We predicted four genes in the transcriptomic that are associated with egg production, namely P2RX1, INHBB, VIPR2, and FABP3, and identified three important pathways during egg production, “Calcium signaling pathway”, “Neuroactive ligand–receptor interaction” and “Cytokine–cytokine receptor interaction”, respectively. In the metabolomic 149 significantly differential metabolites were identified, 99 in the negative model and 50 in the positive model, of which 17α-hydroxyprogesterone, iloprost, spermidine, and adenosine are important metabolites involved in reproduction. By integrating transcriptomics and metabolomics, the correlation between specific differential genes and differential metabolites identified important gene-metabolite pairs “VIPR2-Spermidine” and “P2RX1-Spermidine” in egg production. In conclusion, these data provide a better understanding of the molecular differences between the ovaries of low- and high-production hens and provide a theoretical basis for further studies on the mechanics of poultry egg production.

## 1. Introduction

Eggs are a high-quality protein source for humans and a vital food resource on a global scale. The ovary is a pivotal part of the bird reproductive system and is strongly associated with egg production. Ovarian development is a highly complicated process that involves a plethora of endocrine, autocrine, and paracrine components [1]. Increasing egg production capacity is an essential breeding goal, and traditional breeding approaches can provide good genetic progress in improving chicken egg production, but at a sluggish rate, and the amount of genetic improvement in each generation is difficult to determine [2].

Transcriptomics sequencing technology gives biologically whole transcriptional information at the single nucleotide level [3]. Currently, there is a widespread use of transcriptomics sequencing to identify candidate genes and pathways related to egg production in the poultry ovary [4]. However, transcriptomics sequencing can only provide information at the genetic level and cannot investigate the true level of metabolism in an organism, which makes it difficult to identify the key pathways responsible for regulating specific traits. Meanwhile, metabolomics is closely related to phenomics and can provide a more direct and accurate reflection of the physiological state of an organism [5,6]. For example, Yuan et al. reported the findings of a metabolomic investigation of Stearoyl-CoA desaturase (SCD) during goose follicle development, identifying cholesterol and pantothenol or pantothenate as prospective metabolite biomarkers for the study of SCD-related lipid metabolism in goose granulosa cells (GC) [7]. Therefore, using metabolomics and transcriptomics to study ovarian function in poultry provides a more comprehensive understanding of the underlying mechanisms of egg production. Integration of transcriptomic and metabolomic large-scale datasets has been successfully applied in several animals. A comprehensive analysis of the transcriptomics and metabolomics by Zhan et al. revealed a complex molecular regulatory network for the quality of Enshi black pork [8]. Sun et al. reported the integration of metabolomics with transcriptomics to reveal subtle hepatic metabolic risks in cows fed different crop by-products [9].

The Ninghai indigenous chicken (NH) is a native chicken of Zhejiang Province, which is used for both meat and eggs, with good egg production and peak egg production, and excellent meat value. The Wuliangshan black-boned chicken (WL) is a native breed of Yunnan Province, having meat and chicken egg of excellent meat quality, but low egg production performance [10]. Therefore, the difference in egg production performance between WL and NH could be a suitable model for discovering the potential molecular mechanisms involved in egg production. In this study, we used transcriptomics and metabolomics analyses of different laying hen ovaries, and these findings will provide new insights into the molecular mechanisms of egg production in poultry ovaries and highlight the significance of an integrated approach for this research.

## 2. Materials and Methods

### 2.1. Ethical Statement

All the chickens used in this experiment were handled following the Chinese Animal Welfare Guidelines and as approved by the Animal Welfare Committee of Zhejiang University (approval number: ZJU20190149).

### 2.2. Animal and Sample Collection

Chickens of both breeds were hatched in the same batch, and after hatching the hens were kept and managed under the same conditions by Ningbo Zhenning Animal Husbandry Co., Ltd. in Zhejiang Province, giving them free access to feed and water. Vaccination followed poultry vaccinations established by local animal husbandry and veterinary authorities. At 204 days of age, five healthy hens of each breed were randomly selected and their ovarian tissues were obtained, rinsed in PBS, and quickly frozen in liquid nitrogen.

### 2.3. Metabolite Extraction, Detection, and Analysis

Ovarian tissue (100 mg) was ground separately in liquid nitrogen and subsequently resuspended in pre-chilled methanol (80%) and formic acid (0.1%). Samples were incubated on ice for 5 min and then centrifuged at 15,000 rpm for 5 min at 4 °C. A part of the supernatant was diluted to a concentration containing 60% methanol and transferred to a new centrifuge tube for 10 min at 15,000× *g* at 4 °C. Subsequently, the filtrate was injected into the LC-MS/MS system for analysis. The Vanquish UHPLC system (Thermo Fisher, Waltham, MA, USA) and Orbitrap Q Exactive HF-X mass spectrometer (Thermo Fisher, Waltham, MA, USA) were used to perform the LC-MS/MS analysis as follows: the experimental samples were injected into a Hyperil Gold column with linear gradient time and flow rate set to 16 min and 0.2 mL/min, respectively. Eluent A (0.1 % FA, water) and B (methanol) were used for the positive polarity mode eluent. The negative polarity mode eluent was A (5 mM ammonium acetate, pH 9.0) and B (methanol). Solvent gradients: 2% B for 1.5 min; 2–100% B for 12.0 min; 100% B for 14.0 min; 100–2% B for 14.1 min; 2% B for 16 min. Q Exactive HF-X mass spectrometer spray voltage was set at 3.2 kV, temperature at 320 °C, the intrathecal gas flow rate at 35 arb, and auxiliary gas flow rate at 10 arb.

We used Compound Discoverer 3.0 (CD 3.0, Thermo Fisher, Waltham, MA, USA) to match peaks, take peaks, and quantify each metabolite from the raw data files obtained by UPLC-MS/MS. After normalizing the data, it was utilized to forecast molecular formulae based on addition ions, molecular ion peaks, and fragment ions. The peaks were then compared to the mzCloud (https://www.mzcloud.org/, accessed on 20 August 2020) and ChemSpider (http://www.chemspider.com/, accessed on 20 August 2020) databases to obtain precise qualitative and relative quantitative results.

### 2.4. RNA Sequencing (RNA-Seq) and Data Analysis

The RNA sample preparations used a total of 3 µg of RNA as input material for each sample. All sequencing libraries were prepared using NEBNext^®^ UltraTM RNA Library Prep Kit for Illumina^®^ (NEB, San Diego, CA, USA). In brief, mRNA was isolated from total RNA employing poly-T oligo-attached magnetic beads. Fragmentation was performed out in the NEBNext First Strand Synthesis Reaction Buffer (5X) by divalent cations at elevated temperatures. First strand cDNA was produced using a random hexamer primer and M-MuLV Reverse Transcriptase (RNase H-). Subsequently, second strand cDNA synthesis was carried out with DNA Polymerase I and RNase H. Polymerase activities were used to transform the remaining overhangs into blunt ends. NEBNext Adaptor with hairpin loop structure was ligated after adenylation of 3′ ends of DNA fragments to provide for hybridization. The library fragments were purified using the AMPure XP technology to select cDNA segments preferably 250,300 bp in length. Then, 3 µL of USER Enzyme was used with size-selected, adaptor-ligated cDNA at 37 °C for 15 min, followed by 5 min at 95 °C before PCR. The PCR was then performed using High-Fidelity DNA Polymerase, PCR primers, and In-dex (X) Primer. Finally, PCR products were purified using the AMPure XP system, and library quality was determined using the Agilent Bioanalyzer 2100 system.

Raw fastq data were initially processed using in-house Perl scripts. Clean data were acquired in this stage by eliminating adapter-containing reads, ploy-N, and low-quality reads from raw data, at the same time the clean data’s Q20, Q30, and GC contents were determined [11]. All downstream analysis relied on clean, high-quality data. We used the Hisat2 v2.0.5 to build an index of the reference genome and aligned pairs of clean reads to the reference genome using Hisat2 v2.0.5 [12]. The featureCounts v1.5.0-p3 was used to count the number of reads mapped to each gene [13]. The FPKM of each gene was then computed based on its length and the number of reads mapped to it [14].

We performed differential expression analysis using the R package DESeq2 (1.16.1) and the resulting *p*-values were adjusted using the method of Benjamini and Hochberg to control for false discovery rates. Genes identified by DESeq2 with adjusted *p*-value < 0.05 were designated as differentially expressed. Subsequently, after adjusting for normalization factors for each sequencing library, we performed differential expression analysis for both conditions using the edgeR package (3.18.1) [15]. The *p*-values were adjusted using the Benjamini and Hochberg approach [16]. A corrected *p*-value of 0.05 and an absolute fold change of two conditions was set as the threshold for significant differential expression.

### 2.5. GO and KEGG Enrichment Analysis of DEGs

The clusterProfiler R program was used to perform Gene Ontology (GO) and KEGG enrichment analysis of differentially expressed genes.

### 2.6. Statistical Analysis

The egg production traits of the high- and low-production groups were compared by *t*-test. The significance level in the analyses was considered at *p* < 0.05. The analysis and plotting were performed using RStudio software.

## 3. Results

### 3.1. Production Performance

**Figure 1:**
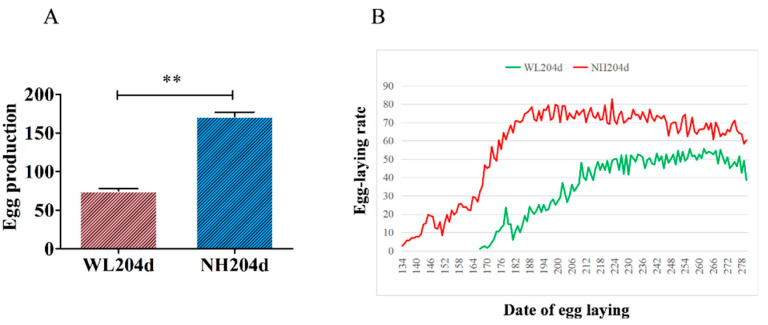
Analysis of egg production of different breeds of hens. (**A**) comparison of the average number of eggs laid by two breeds of hens over a statistical number of days. Data is shown as mean ± standard deviation (SD), ** indicates *p* < 0.01. (**B**) graphs of egg production rates of different breeds of hens, with horizontal coordinates representing days of laying and vertical coordinates representing egg production rates. The red curve represents the egg production rate of NH (*n* = 300). The green curve represents the laying rate of WL (*n* = 200).

In this study, the number of eggs produced by the two breeds of hens at 280 days was counted and the egg production rate graphs were plotted, which showed that the difference in egg production between the two breeds of hens was extremely significant (as shown in Figure 1).

### 3.2. Quality Control, Principal Component Analyses of Metabolomics

**Figure 2:**
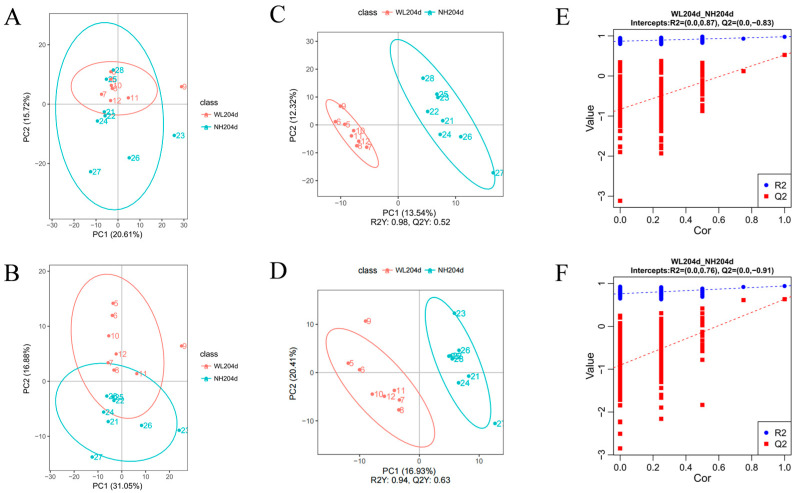
Metabolome quality control analysis. (**A**) PCA analysis in the negative model; (**B**) PCA analysis in the positive model; (**C**) PLS-DA analysis in the negative model; (**D**) PLS-DA analysis in the positive model; (**E**) permutation test of PLS-DA in the negative model; (**F**) permutation test of PLS-DA in the positive model.

In this study, metabolomics analyses were performed on ovarian tissues from hens in the high- and low-production groups to detect overall biochemical changes (*n* = 8). Two-dimensional principal component analysis score plots showed that the overall trends in metabolite distribution differed between samples from the high- and low-production groups in the negative model (Figure 2A) and the positive model (Figure 2B). In addition, a clear separation of metabolites between the high and low egg production groups was observed in the negative model (Figure 2C) and the cation model (Figure 2D) by plotting in the PLS-DA score, with the permutation test confirming the accuracy of PLS-DA models (Figure 2E,F).

### 3.3. Metabolomics Analysis of High- and Low-Production Groups

**Figure 3:**
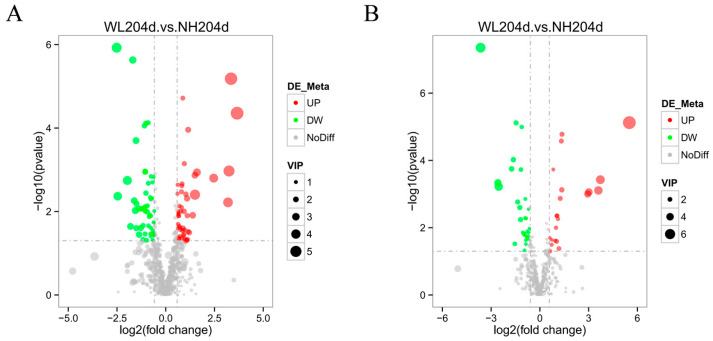
Volcano plots of differential metabolites in the ovaries of hens with different egg production rates. Red represents significantly up-regulated differential metabolites, green indicates significantly down-regulated differential metabolites. (**A**) Differential metabolites in the negative model; (**B**) Differential metabolites in the positive model.

To identify the significantly differential metabolites (SDMs) between high- and low-production groups, the Variable Importance in the Projection (VIP) of the first component of the PLS-DA model was used, and the significantly differential metabolites were found by combining the *p*-value of the *t*-test. A total of 149 significantly different metabolites (99 negative model and 50 positive model) were identified by setting thresholds VIP > 1, difference fold change (FC) >1.5 or FC < 0.667, *p*-value < 0.05 (Supplementary Table S1). The negative and positive model volcano graphs for the differential metabolites are shown in Figure 3, with each point in the diagram representing an identified metabolite.

### 3.4. KEGG Analysis of SDMs

**Figure 4:**
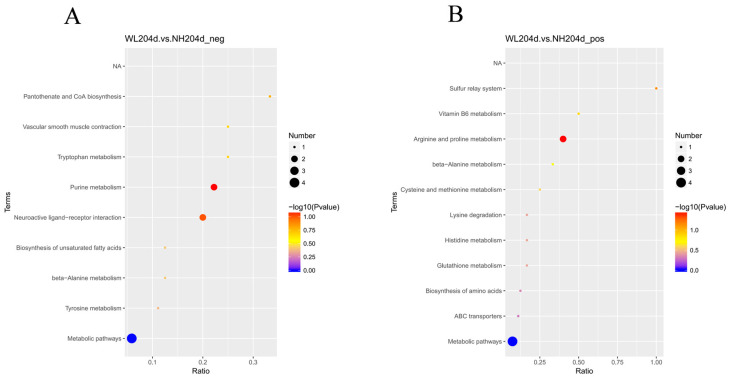
KEGG enrichment analysis for SDMs. (**A**) KEGG enrichment of significantly differential metabolites in the negative model; (**B**) KEGG enrichment of significantly differential metabolites in the positive model.

KEGG database annotations were set as background, and all the SDMs were subjected to KEGG annotation analysis; 20 pathways were identified including nine terms in the negative model and 11 terms in the positive model. KEGG enrichment analysis showed that differential metabolites in the two groups were mainly involved in “metabolic pathways”, “arginine and proline metabolism”, “purine metabolism”, and “neuroactive ligand–receptor interaction” (Figure 4, Supplementary Table S2).

### 3.5. Transcriptomics Analysis of High- and Low-Production Groups

**Figure 5:**
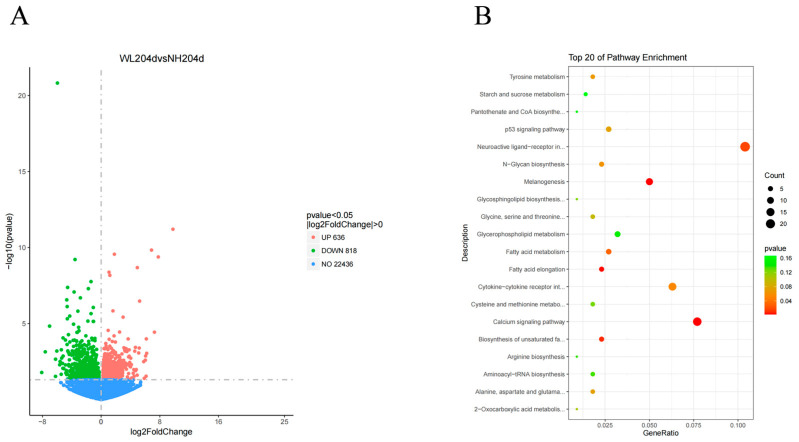
Bioinformatics analysis of hens with different egg production rates. (**A**) The volcano plot of differential genes in the ovaries of hens with different egg production rates. The x-axis represents the log2 fold change, the y-axis represents the statistical significance. The red dots indicate significantly up-regulated differential genes, the green dots indicate significantly down-regulated differential genes and the blue dots indicate no significant differential genes; (**B**) The top 20 KEGG terms presented in the enrichment analyses of ovary DEGs between high- and low-production hens.

Eight cDNA libraries were constructed for the ovaries of low- and high-production chickens, obtaining 68.86 GB of clean reads, including 459,114,490 reads generated after quality control assessment, with an average of 57,389,311.25 per group (Table 1). A total of 1454 DEGs (differentially expressed genes) were identified between the low- and high-production groups (*p*-value < 0.05 and |log2FC| > 0). Compared with the high-production group, the low-production group showed 636 and 818 up- and down-regulated genes, respectively (Figure 5A, Supplementary Table S3). All DEGs were mapped to KEGG pathways and obtained 125 enriched pathways, and we listed the top 20 KEGG pathways (Figure 5B, Supplementary Table S4), among which “Melanogenesis”, “Calcium signaling pathway” and “Neuroactive ligand–receptor interaction” and “Cytokine–cytokine receptor interaction” were the most enriched pathways in the up- and down-regulated groups. The “Calcium signaling pathway” and “Neuroactive ligand–receptor interaction” were the two most representative pathways.

### 3.6. Comprehensive Analysis of the Transcriptomics and Metabolomics

**Figure 6:**
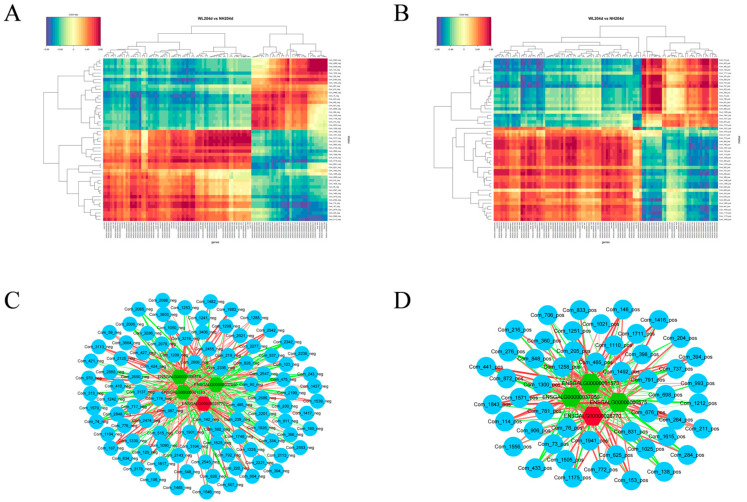
Correlation heat maps and network plots of DEGs and SDMs. (**A**) Heat map of the correlation between significantly differential genes and significantly differential metabolites in the negative model; (**B**) Heat map of the correlation between significantly differential genes and significantly differential metabolites in the positive model; (**C**) Correlation network diagram in the negative model; (**D**) Correlation network diagram in the positive model. Circles indicate differential metabolites, hexagons indicate differential genes, red indicates up-regulated genes, green indicates down-regulated genes, red lines indicate positive correlations, green lines indicate negative correlations and the thickness of the line indicates the strength of the correlation.

Correlation analysis utilized Pearman calculations to show the correlation of the DEGs from transcriptomics and the SDMs from metabolomics. When the correlation coefficient is less than 0, it is referred to as a negative correlation; when it is greater than 0, it is referred to as a positive correlation. The top 50 differential metabolites and the top 100 differential genes are shown. Results showed strong correlations between transcripts and metabolites (Figure 6, Supplementary Table S5). In addition, we correlated specific genes that potentially regulate egg production with metabolites, looking for important gene–metabolites to explore further potential pathways of action (Figure 6). We considered correlations > 0.8 and *p*-values < 0.05 as strongly correlated gene–metabolite pairs and plotted the network using Cytoscape 3.8.0.

**Figure 7:**
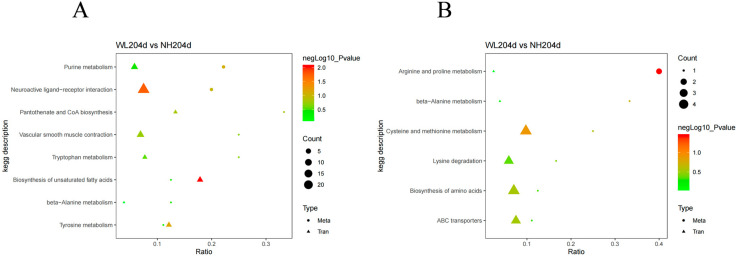
Common KEGG pathway enrichment map of the transcriptome and metabolome. (**A**) The common pathways in the negative model; (**B**) The common pathways in the positive mode.

DEGs in the transcriptome and SDMs in the metabolome were enriched into a total of 13 pathways. The pathways that were co-enriched in the negative model were “Pantothenate and CoA biosynthesis”, “Tyrosine metabolism”, “Vascular smooth muscle contraction”, “Biosynthesis of unsaturated fatty acids”, “Neuroactive ligand-receptor interaction”, “beta-Alanine metabolism”, “Tryptophan metabolism”, and “Purine metabolism”. The pathways coenriched in the positive model were “ABC transporters”, “Arginine and proline metabolism”, “Lysine degradation, beta-Alanine metabolism”, “Biosynthesis of amino acids”, “Cysteine and methionine metabolism”, However, there were no significantly enriched common pathways among all the pathways (Figure 7, Supplementary Table S6).

## 4. Discussion

Egg production is an important indicator of poultry reproductive performance, and the ovary is an organ closely related to egg production. An intensive study of ovarian mechanisms will provide a better understanding of the egg production mechanism of the poultry ovary. In this study, we used transcriptomics and metabolomics to investigate the mechanism of egg production and to investigate the ovaries of chickens with different egg production rates to provide new insights into the egg production mechanisms of poultry in a more comprehensive way and to provide new data to support future research on egg production performance.

### 4.1. Transcriptomics Analysis

In this study, we identified 1454 DEGs in the ovaries of hens with different egg production rates, of which 636 genes were up-regulated and 818 genes were down-regulated. These results suggested that these DEGs may play an important role in egg-laying regulation. A total of 125 pathways were revealed in the ovary, with “neuroactive ligand–receptor interaction” having the most DEGs, followed by “calcium signaling pathway” and “cytokine–cytokine receptor interaction”. The “neuroactive ligand–receptor interaction” pathway plays an essential role in egg production in chickens, which is consistent with a study on the Jinghai Yellow chicken [17]. Previous studies have suggested that “neuroactive ligand–receptor interaction” may be the most important pathway leading to differences in egg production rates between high- and low-production hens; in addition, the pathway is proposed to be involved in the regulation of egg-laying performance in ducks [18] and geese [19]. Calcium (Ca^2+^) is an essential signaling molecule that controls a wide range of biological functions, and the calcium signaling pathway is associated with egg production in poultry [20,21]. Zhang et al. [22] found the pathway correlated with eggshell quality in chickens. The high DEGs in the “cytokine–cytokine receptor interaction” in this study coincided with reports on egg production in Nandan-Yao domestic chicken and Muscovy duck [23,24]. In brief, the three pathways, “neuroactive ligand–receptor interaction”, “calcium signaling pathway”, and “cytokine–cytokine receptor interaction” are considered to be closely related to chicken egg production performance.

P2RX1 is one of the “calcium signaling pathway” enriched genes and subtypes of the P2X1 receptor [25]. Calcium signaling plays an important role in animal development and reproduction, and lack of P2X1 affects sperm transport in male mice leading to impaired reproductive function [26]. The gene has also been reported as a potential regulator of ovarian egg production in white ducks [24]. In this study, the expression of this gene was significantly higher in hens of the high egg-laying group than in the low egg-laying group, and it is hypothesized that this gene has an important role in the egg-laying process of hens. INHBB (inhibin beta B subunit) is a glycoprotein hormone that belongs to the transforming growth factor-β superfamily and inhibits follicle-stimulating hormone (FSH) production and secretion [27]. Knockdown of INHBB increases apoptosis and inhibits steroidogenesis in mouse granulosa cells [28], and the gene was also found to be closely associated with reproductive processes in sheep [29]. High expression of INHBB transcripts in the ovaries of WL hens may increase the apoptosis of granulosa cells, inhibit hormone production, and lead to lower egg production. VIPR2 (vasoactive intestinal peptide receptor 2) belongs to the VIP/ PACAP type II receptor, also named the pituitary adenylate cyclase-activating polypeptide (PACAP) receptor. PACAP is a biologically active peptide transiently expressed in the preovulatory follicle that stimulates ovarian function [30]. In a previous report, this gene was found to be associated with granulosa cell proliferation and apoptosis in the ovary [21]. However, Sun et al. found that it may be at the GWF developmental stage and that high VIPR2 expression may be associated with brooding and lower egg-laying traits in hens [31]. In this study, it was found to have higher expression in high egg-laying ovaries and may have a positive effect on egg production. The function of this gene remains to be further investigated. FABP3 (fatty acid binding protein 3) encodes a fatty acid transporter of long-chain fatty acids (LCFA) and is related to the PPAR/RXR signaling pathway [32]. LCFA has an essential role in the regulation of energy metabolism [33]. FABP3 was significantly higher in the high laying group than in the low laying group, presumably requiring a higher energy supply to prepare the eggs during laying.

### 4.2. Metabolomics Analysis

Metabolomics, a relatively new field that emerged in response to genetics and proteomics, can illustrate the physiological state of an organism by monitoring changes in endogenous metabolites. Currently, metabolomics is widely used in various fields in a wide range of animals. In this study, we discovered 149 substantially different compounds in the ovaries of chickens with different egg production rates, 50 in the positive mode and 99 in the negative mode, under a self-constructed database.

The three pathways “cysteine and methionine metabolism”, “arginine and proline metabolism” and “glutathione metabolism” may be involved in the egg production process of hens. Feng et al. found that arginine improved the reproductive performance of hind sows by adding arginine (ARG) to the diet [34]. There is evidence that ovarian glutathione is an important defense against oxidative damage, that glutathione in oocytes is essential in early embryonic development, and that glutathione biosynthesis is heavily dependent on the metabolism of cysteine and methionine [35,36]. Among the significantly different metabolites, the synthetic steroid hormone hydroxyprogesterone caproate, which is comparable to megestrol acetate and medroxyprogesterone acetate, is an ester derivative of 17-hydroxyprogesterone produced from caproic acid (hexanoic acid). In previous studies, it was discovered to delay the onset of preterm birth [37]. Iloprost is a PGI2 analogue, prostacyclin (PGI2), which is synthesized in the oviductal fluid and promotes embryonic development before implantation. Iloprost can alter the maturation rate of bovine oocytes and the expansion of the oocyte [38]. The addition of iloprost to the maturation medium improved the developmental potential and embryonic quality of porcine IVF embryos, including the mitochondrial membrane potential, mRNA expression of apoptosis-related genes, and susceptibility to apoptosis [39]. Spermidine is a polyamine, an aliphatic polymer, expressed in ovarian granulosa cells and follicular membrane cells [40]. It plays an important role in the maintenance of the cellular macromolecular structure, cell growth and proliferation, and the prevention of oxidative stress [41]. The exogenous supplementation of spermidine and spermine in cultures of pig and mouse uterine stroma and epithelial cells upregulates the gene expression of *SAT1* [42]. A lack of polyamines may lead to stalled follicular cell development, which in moderate amounts is the basis of membrane and granulosa cell development in the ovary, but too much spermidine induces ovarian oxidative stress and granulosa cell apoptosis [43]. Adenosine is primarily created by the breakdown of adenosine triphosphate (ATP) and serves a variety of roles throughout the body [44]. Together with some purines, adenosine constitutes an important and fairly common modulator of neuronal activity, and it is a general inhibitor of neuronal activity. A typical physiological effect is inhibiting the release of neurotransmitters. Adenosine is enriched in “neuroactive ligand–receptor interaction” and could act as an essential metabolic marker. In summary, the above significantly differential metabolites may be associated with essential metabolites during egg production in poultry.

### 4.3. Combining Analysis of Transcriptomics and Metabolomics

We correlated specific differential genes with metabolites, analyzed specific genes potentially regulating egg production with correlations and integrated gene-metabolite pairs based on the gene–metabolite correlations, and identified two important gene–metabolite pairs, VIPR2–spermidine and P2RX1–spermidine. VIPR2 and P2RX1 being potentially VIPR2 and P2RX1 are potential egg production regulatory genes with a strong positive correlation to Spermidine, which is an important metabolite in reproduction as described above. Taken together, these two gene metabolites are likely to be essential regulators of egg production in hens.

## 5. Conclusions

In this study, we used transcriptomics and metabolomics to study the ovaries of different breeds of laying hens, discussing the genes that potentially regulate egg production processes including P2RX1, INHBB, VIPR2, and FABP3, as well as the important ovarian metabolites 17α-hydroxyprogesterone, iloprost, spermidine, and adenosine. In addition, we identified two essential metabolite pairs through gene and metabolite association analysis, namely, VIPR2–Spermidine and P2RX1–Spermidine during egg production. These results may contribute to our understanding of the developmental molecular process of egg production in poultry and provide supporting material for future breeding.

## Figures and Tables

**Table 1:** Sequencing data.

Sample	Raw_Reads	Clean_Reads	Total-Map	Error_Rate	Q30	GC_Pct
WL204d_1	59,739,580	58,953,024	54,412,280 (92.3%)	0.02%	94.49%	51.2%
WL204d_2	58,473,150	57,710,008	52,207,089 (90.46%)	0.02%	94.4%	51.23%
WL204d_3	61,333,900	60,429,438	54,577,702 (90.32%)	0.03%	94.07%	51.33%
WL204d_4	57,835,138	56,950,072	51,440,969 (90.33%)	0.03%	94.2%	50.71%
NH204d_1	59,434,470	58,593,266	53,253,424 (90.89%)	0.03%	94.22%	50.91%
NH204d_2	53,435,540	52,503,528	47,500,358 (90.47%)	0.03%	94.22%	50.53%
NH204d_3	62,255,842	61,476,260	56,020,220 (91.12%)	0.03%	94.17%	51.38%
NH204d_4	53,250,580	52,498,894	46,560,294 (88.69%)	0.03%	94.17%	51.69%

## Data Availability

The sequence data has been submitted to the NCBI SRA database under accession number PRJNA798516 and PRJNA798791.

## Supplementary Materials

The following supporting information can be downloaded at: https://www.mdpi.com/article/10.3390/ani12162010/s1, Table S1: Significantly differential metabolites; Table S2: KEGG enrichment analysis of SDMs; Table S3: Significantly differential expression genes; Table S4: All KEGG pathways of DEGs; Table S5: Correlation of DEGs and SDMs; Table S6: The common KEGG pathways of Transcriptomics and Metabolomics.
